# Leukocyte numbers and function in subjects eating *n*-3 enriched foods: selective depression of natural killer cell levels

**DOI:** 10.1186/ar2426

**Published:** 2008-05-14

**Authors:** Violet R Mukaro, Maurizio Costabile, Karen J Murphy, Charles S Hii, Peter R Howe, Antonio Ferrante

**Affiliations:** 1 Department of Immunopathology, Children, Youth and Women's Health Service, 72 King William Road, North Adelaide SA 5006, Australia; 2 Nutritional Physiology Research Centre, School of Health Sciences, University of South Australia, Adelaide SA 5000, Australia; 3 School of Pharmaceutical and Medical Sciences, University of South Australia, Australia, Adelaide SA 5000, Australia; 4 Discipline of Paediatrics, University of Adelaide, 72 King William Road, North Adelaide SA 5006, Australia

## Abstract

**Introduction:**

While consumption of omega-3 long-chain polyunsaturated fatty acids (*n*-3 LCPUFA) has been recommended for those at risk of inflammatory disease such as rheumatoid arthritis, the mechanism of their anti-inflammatory effect remains to be clearly defined, particularly in relation to the dose and type of *n*-3 LCPUFA. The objective of this study was to determine whether varying the levels of *n*-3 LCPUFA in erythrocyte membrane lipids, following dietary supplementation, is associated with altered numbers and function of circulating leukocytes conducive to protection against inflammation.

**Methods:**

In a double-blind and placebo-controlled study, 44 healthy subjects aged 23 to 63 years consumed either standard or *n*-3 LCPUFA-enriched versions of typical processed foods, the latter allowing a target daily consumption of 1 gram *n*-3 LCPUFA. After six months, peripheral blood leukocyte and subpopulation proportions and numbers were assessed by flow cytometry. Leukocytes were also examined for lymphoproliferation and cytokine production, neutrophil chemotaxis, chemokinesis, bactericidal, adherence and iodination activity. Erythrocytes were analyzed for fatty-acid content.

**Results:**

Erythrocyte *n*-3 LCPUFA levels were higher and absolute leukocyte and lymphocyte numbers were lower in subjects consuming *n*-3 enriched foods than in controls. There were no changes in the number of neutrophils, monocytes, T cells (CD3^+^), T-cell subsets (CD4^+^, CD8^+^) and B cells (CD19^+^). However, natural killer (NK) (CD3^-^CD16^+^CD56^+^) cell numbers were lower in *n*-3 supplemented subjects than in controls and were inversely related to the amount of eicosapentaenoic acid or docosahexaenoic acid in erythrocytes. No significant correlations were found with respect to lymphocyte lymphoproliferation and production of IFN-γ and IL-2, but lymphotoxin production was higher with greater *n*-3 LCPUFA membrane content. Similarly, neutrophil chemotaxis, chemokinesis, bactericidal activity and adherence did not vary with changes in erythrocyte *n*-3 LCPUFA levels, but the iodination reaction was reduced with higher *n*-3 LCPUFA content.

**Conclusion:**

The data show that regular long-term consumption of *n*-3 enriched foods leads to lower numbers of NK cells and neutrophil iodination activity but higher lymphotoxin production by lymphocytes. These changes are consistent with decreased inflammatory reaction and tissue damage seen in patients with inflammatory disorders receiving *n*-3 LCPUFA supplementation.

## Introduction

There is evidence from both experimental models and clinical studies that long-chain omega-3 polyunsaturated fatty acids (*n*-3 LCPUFA) are beneficial in the treatment of autoimmune and allergic inflammatory conditions [1-4]. Counterbalancing *n*-6 fatty-acid intake with *n*-3 fatty acids is important because *n*-6 fatty acids, such as arachidonic acid (AA) are released during cellular activation and inflammation and metabolized to generate highly inflammatory metabolites such as the 4-series leukotrienes and 2-series prostaglandins [5-7]. Increasing the amounts of eicosapentaenoic acid (EPA) in membrane phospholipids not only reduces the level of AA available for metabolism by the lipoxygenases and cyclooxygenases, but EPA also competes against AA for metabolism to form metabolites of the 5-series leukotrienes and 3-series prostaglandins, which are significantly less pro-inflammatory than the AA-derived metabolites, thereby taming the inflammatory reaction [4].

The effects of *n*-3 LCPUFA supplementation on inflammation and immune responses have been extensively studied [8]. This includes the benefits in treating patients with rheumatoid arthritis (RA), Crohn's disease, ulcerative colitis, systemic lupus erythematosus, psoriasis, atherosclerosis and asthma [9]. Despite the finding of such beneficial effects there is still insufficient evidence to enable specific recommendations to be made on the use of *n*-3 fats in these disorders [10]. These include knowledge of efficacious doses of *n*-3 fatty acids and the type of *n*-3 fat that is most effective. The evidence indicates that EPA and docosahexaenoic acid (DHA) have differential effects which may be further complicated by the array of immune cells and pathways which can be altered by these PUFAs [5]. This would explain the variation from study to study in the degree of benefit attained for different conditions.

Here we examine the effects of providing low-dose long-term *n*-3 fatty acids in foods on a number of parameters of innate and adaptive immune response. We also took this opportunity to explore the relationship between membrane fatty-acid composition and several components of the immune system relevant to inflammatory diseases such as RA by analyzing leukocyte levels and functional responses in blood samples obtained from subjects receiving *n*-3 LCPUFA supplementation in a 6-month intervention trial. The results showed that a higher level of *n*-3 LCPUFA in erythrocyte membrane phospholipids is associated primarily with a significantly lower number of circulating natural killer (NK) cells, which could be considered beneficial in reducing tissue damage in chronic inflammatory diseases.

## Materials and methods

Ficoll 400 was obtained from Pharmacia Biotech (Uppsala, Sweden). RPMI 1640 medium and glutamine were obtained from JRH Biosciences (Lenexa, KA). Sodium diatrizoate, DMSO, *N*-formyl-methionyl-L-leucyl-L-phenyalanine (fMLP), Rose Bengal stain (0.25 % w/v in phosphate-buffered saline (PBS) with Ca^2+^, Mg^2+^), zymosan and 3,3',5,5'-tetramethylbenzidine were purchased from Sigma (St Louis, MO). Angiograffin was obtained from Schering (Leverkusen, Germany). Agarose was purchased from Calbiochem (La Jolla, CA). Tumor necrosis factor (TNF) was obtained from the Ernst-Boehringer Ingelheim Institut (Vienna, Austria). The *Staphylococcus aureus *strain NCTC 6571, for measuring neutrophil bactericidal activity, was obtained from the National Centre for Tissue Cultures (Oxford, UK). ^125^I in the form of NaI and [H^3^]thymidine was purchased from Amersham International (Little Chalfont, UK). All monoclonal antibodies for the determination of lymphocyte subsets, IgG1 isotype control antibody and Simultest IMK kit were obtained from BD Biosciences (San Jose, CA). Phytohemagglutinin (PHA) was obtained from Murex Diagnostics (Dartford, UK). Anti-human interferon-γ (IFNγ) coating monoclonal antibody and anti-human interleukin-2 (IL-2) polyclonal antibody were purchased from Endogen (Rockford, IL). The biotin-labeled anti-human IFNγ and anti-human IL-2-detecting monoclonal antibodies, horseradish peroxidase, streptavidin and quality-control sample for IFNγ were also purchased from Endogen. The anti-human lymphotoxin (LT) coating and biotin-labeled anti-human LT-detecting monoclonal antibodies were purchased from R&D Systems (Minneapolis, MN). The quality-control samples for LT and IL-2 and standards for IFNγ were obtained from the National Institute for Biological Standards and Control (South Mimms, UK). The standards for LT were purchased from Biosource International (Camarillo, CA). The standards for IL-2 were purchased from Hazelton Biotechnologies (Vienna, Austria).

### Study foods

A range of study foods including pancake mix, bread, milk, margarine, eggs, chocolate, soup mix, dips, instant oats, cheese spread, muffin mix, biscuits and salad dressing were provided by Goodman Fielder (Sydney, Australia). Foods were either enriched with *n*-3 LCPUFA from microencapsulated cod liver oil (Maritex, Aarhus, Denmark) (*n*-3 supplemented) or were devoid of *n*-3 LCPUFA (placebo). The fatty-acid composition of the study foods is described elsewhere [10,11]. Each enriched food portion provided 125 mg EPA + DHA and subjects were asked to consume eight exchanges daily, to equal 1 g *n*-3 PUFA/day. Subjects were matched for gender and age then randomly allocated to treatment or control groups. Dietary interviews were conducted by trained dieticians using diet questionnaires and food records as described by Patch *et al*. [10] to score the acceptability and palatability of individual food items to ensure compliance with test foods. Subjects were encouraged to keep 'diet diaries' in order to monitor the amount of *n*-3 PUFA-rich foods consumed. The target macronutrients intakes (% of energy) were as follows: 20% protein, 50% carbohydrate, 30% fat (polyunsaturated: mono-unsaturated: saturated = 1:1:1) [11].

### Subjects and study design

A double-blind, placebo-controlled dietary intervention study of 6 months duration was approved by the Human Research Ethics Committees of the University of Adelaide and the Commonwealth Scientific and Industrial Research Organization (CSIRO) (see Murphy *et al*. [11] for details). The trial commenced in 2003 and consent from the subjects recruited was obtained. Forty-four non-smoking volunteers aged 20 to 65 years, who were overweight (BMI > 25 kg/m^2^) and had fasting plasma triacylglycerol > 1.6 mmol/l, but were otherwise healthy, were recruited from the general community through media advertisements. Volunteers were excluded if they were taking non-steroidal anti-inflammatories, antihypertensives, lipid-lowering or other drugs affecting lipid metabolism. Other exclusion criteria include the consumption of more than one fish meal per week, regularly taking fish oil supplements, inability to consume test foods, recent (previous 3 months) diagnosis of diabetes, symptomatic heart disease, angina pectoris, history of myocardial infarction or stroke, peripheral vascular disease, liver or renal disease (plasma creatinine > 120 mmol/l), major surgery, blood pressure (BP) > 170/100 mmHg; or smokers and/or ex-smokers within the past two years.

### Erythrocyte membrane fatty-acid composition

The fatty-acid composition was analyzed at 6 months as described by Murphy *et al*. [11]. Erythrocytes were isolated, lysed and the membrane lipids extracted into methanol: toluene (4:1, by volume) according to the method of Lepage and Roy [12]. Fatty-acid methyl esters were analyzed by flame-ionization gas chromatography (model GC-20A, Shimazdu Corporation, Kyoto, Japan) using a 50-m BPX70 capillary column (0.32 mm internal diameter and 0.25 μm film thickness (Scientific Glass Engineering, Melbourne, Australia). Individual fatty acids were identified by comparison with known fatty-acid standards (Nuchek Prep, Elysian, MN) and expressed as a percentage of total fatty acids quantified from peak areas.

### Preparation of peripheral blood mononuclear cells and neutrophils

At the end of the 6-month trial, 10 ml blood was collected by venepuncture after a 12-h overnight fast into lithium heparin tubes. Mononuclear cells (MNL) and neutrophils were purified by the rapid one-step procedure according to Ferrante and Thong [13]. Briefly, heparinized blood was layered onto a density separation medium consisting of Ficoll 400-Hypaque, density 1.114. Following centrifugation at 600 *g *for 35 min, the MNL and neutrophil bands were harvested and washed twice with RPMI 1640 medium by centrifugation (5 min × 600 *g*) and MNL resuspension in RPMI 1640 medium or neutrophils in Hanks Buffered Salt Solution (HBSS).

### Neutrophil chemotaxis and chemokinesis

Neutrophils, 5 μl of 4 × 10^7^cells/ml, were allowed to migrate under agarose for 90 min at 37°C/5% CO_2_-air incubator as previously described [14] in the presence of a chemotactic gradient generated by 5 μl 10^-7 ^M of fMLP, or 5 μl of diluent DMSO (1% v/v in PBS). The distance migrated by the cells was expressed in mm/90 min. For chemokinesis, neutrophils (20 μl; 4 × 10^7^/ml) were pre-incubated with 20 μl fMLP for 5 min at 37°C in a humidified CO_2 _(5% CO_2 _in air) incubator, centrifuged in a microcentrifuge (30 sec × 1 *g *force), the supernatant was removed and the cells resuspended in 20 μl HBSS with phenol. The random migration in four directions – top, bottom, left and right – was measured using an inverted Leitz microscope. Chemokinesis was expressed as the mean of the distance (in millimeters) traveled in the four directions.

### Neutrophil adherence

Neutrophil adherence was assayed by measuring neutrophil adherence to plasma-coated plastic surfaces [15]. To the 96-well microtiter plates was added 250 μl/well of 10% autologous plasma and the plates were incubated for 30 min 37°C in a humidified CO_2 _(5% CO_2 _in air) incubator. The plates were subsequently washed with HBSS and air-dried. Then, 100 μl of 5 × 10^6 ^neutrophils treated with either TNF or with HBSS as a control were loaded into the wells and incubated for 30 min at 37°C in a CO_2 _incubator. Non-adherent cells were removed by inversion of plates and the wells washed three times with HBSS. Adherent cells were stained with Rose Bengal (0.25 % w/v in PBS with Ca^2+^, Mg^2+^) [14], washed and the dye was then released by adding 50% ethanol and the absorbance was read at 570 nm using a plate reader (Dynatech MR7000, Dynatech Laboratories, Chantilly, VA). The degree of adherence was calculated by subtracting the mean absorbance values of blank wells from the mean of the test wells.

### Neutrophil bactericidal activity

The ability of neutrophils to kill *Staphylococcus aureus *was assessed as described previously [16]. Neutrophils (1 × 10^6 ^cells) were mixed with 1 × 10^6 ^*S. aureus *in HBSS and 10% (final) pooled human AB serum in tubes which were then gassed with 5% CO_2_-air mixture and incubated with end-to-end mixing at 37°C. Samples were taken at 0, 1 and 2 h, diluted in sterile distilled water and plated onto blood agar for colony growth and enumeration of the number of surviving bacteria.

### Neutrophil iodination reaction

The quantitative neutrophil iodination reaction, which examines the ability to produce oxygen radicals and the release of myeloperoxidase enzyme, was determined by the method described by Thong and Ferrante [17]. Briefly, 25 μl of ^125^I (200 μCi/ml) was added to 1 ml pooled human serum (1:4 dilution in HBSS) and 25 ml added to appropriate wells in a 96-well microtiter plate. Six wells were used for either pooled serum or autologous serum, three of which were stimulated with 50 μl zymosan. As a control, 50 μl of HBSS was added to the remaining three. The plates were incubated for 30 min at 37°C in a humidified CO_2 _(5% CO_2 _in air) and then 50 μl of 1 × 10^7 ^neutrophils/ml were added to appropriate wells. The plates were covered and incubated for 90 min. Finally, the cells were harvested using a cell harvester (Titertek Cell Harvester 550) and the quantity of bound ^125^I was expressed as picomoles/10^7 ^neutrophils. The amount of iodination due to stimulation was calculated by subtracting the basal iodination value (no zymosan added) from the stimulated iodination value (plus zymosan).

### Lymphocyte phenotyping and leukocyte numbers

Lymphocyte subpopulations were determined using a lymphocyte kit and direct two-color immunofluorescence. The kit allows for determination of all T cells (CD3^+^/CD4^+ ^and CD3^+^/CD8^+^), monocytes (CD16^+^), NK cells (CD3^-^/CD16^+/^56^+^) and B lymphocytes (CD19^+^) in one sample simultaneously. One hundred microliters of whole-blood samples were mixed with 2 ml of 1× lysing solution, vortexed and incubated for 10 min at room temperature in the dark. Immediately after incubation the tubes were centrifuged for 5 min at 300 *g*, the supernatant was removed and the pellet resuspended in 2 ml Isoton II. The cells were then centrifuged (300 *g *× 5 min), the supernatant was removed and the pellet fixed in formaldehyde (200 μl 1% solution). The fluorescence intensity was measured using a flow cytometer (FACScan, BD Biosciences, NSW, Australia). The data was processed with Lysis II software (BD Biosciences). To determine leukocyte and lymphocyte numbers, 130 μl of whole blood was aspirated and analyzed using a hematology analyzer CELL-DYN 3500SL (Abbott Diagnostics, North Chicago, IL).

### Measurement of lymphoproliferation

Mononuclear cells in samples of 100 μl (2 × 10^5 ^cells) were cultured in medium supplemented with 4 mM L-glutamine solution, 100 U/ml penicillin, 100 μg/ml streptomycin, 5% human heat-inactivated AB serum and 100 μl PHA (2 μg/ml). The final volume of the culture was 200 μl, and all cultures were performed in triplicate as described previously [18]. Proliferation was measured as the incorporation of [^3^H]thymidine over the final 6 h of a 72-h culture period.

### Measurement of cytokine production

Mononuclear cells were cultured in concentrations and conditions as described above, the culture medium was removed after 72 h and stored at -70°C for cytokine (LT, IL-2 and IFNγ) analysis by enzyme-linked immunosorbent assay [18].

### Statistical analysis

All statistical analyses were performed using GraphPad InStat software. Data were analyzed as comparisons between placebo and *n*-3 PUFA-supplemented groups, as well as correlations between the membrane fatty-acid levels and specific immunological parameters. The Kolmogorov-Smirnov test was used to determine normal distribution of data. Linear regression analyses were performed and statistical comparisons were carried out using Student's two-tailed *t*-test for paired or unpaired data and *p *< 0.05 was considered significant.

## Results

### Erythrocyte membrane fatty-acid composition

**Figure 1:**
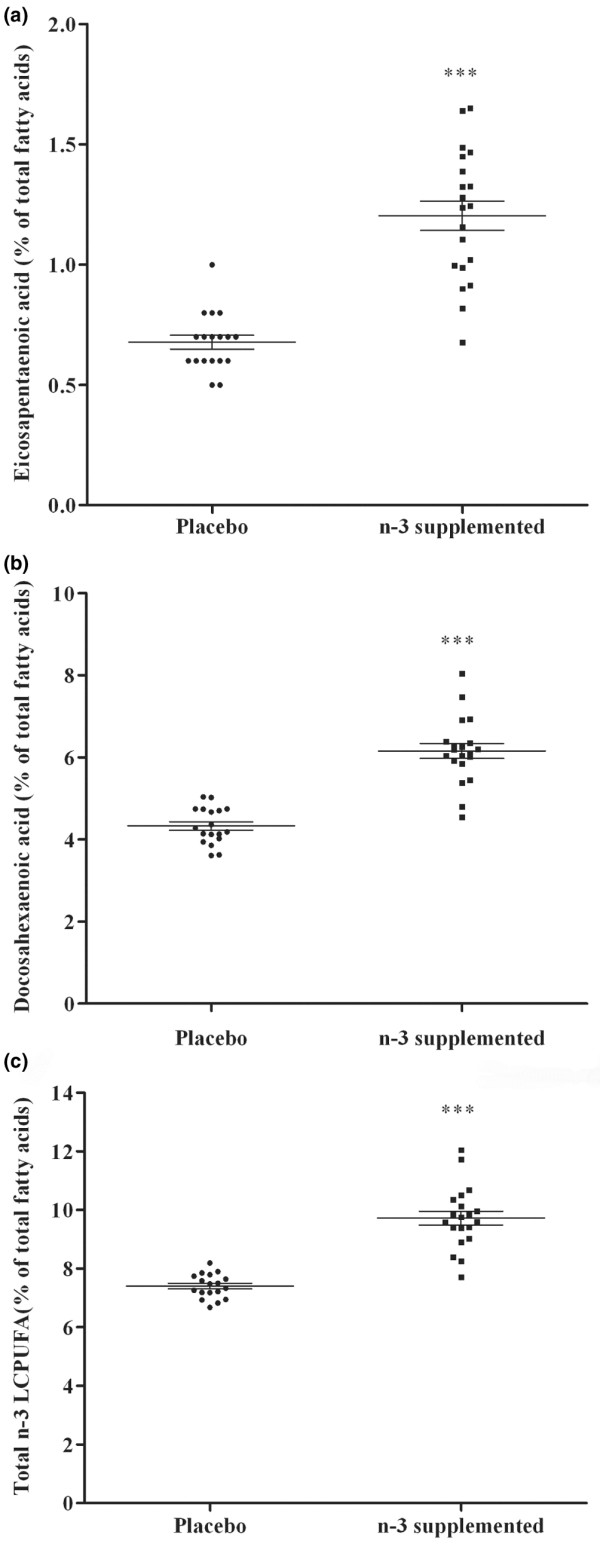
Levels of *n*-3 fatty acids in membrane phospholipids of erythrocytes. **(a) **Eicosapentaeonic acid (EPA), **(b) **docosahexaenoic aid (DHA) and **(c) ***n*-3 LCPUFA (EPA + docosapentaenoic acid (DPA) + DHA) in the placebo and supplemented groups. Blood was taken after 6 months and analyzed for fatty-acid composition as outlined in Materials and methods. Data are expressed as mean ± SEM; *n *= 18 for the placebo group and *n *= 20 for the supplemented group. ***p < 0.0001, Student *t*-test.

A total of 42 (21 placebo and 21 supplemented) individuals completed the study; however, a few samples were not viable and/or were lost, thus accounting for the variation in sample numbers. Analysis of the erythrocyte membrane lipid composition (*n *= 18 placebo and *n *= 20 *n*-3 LCPUFA supplemented) showed considerable overlap in levels of *n*-3 and *n*-6 PUFA, EPA and DHA between the two groups (Figure 1). The EPA, DHA and total *n*-3 LCPUFA (docosapentaenoic acid (DPA) + EPA + DHA) content in the enriched group was significantly higher than in the placebo group (Figure 1), whereas the total *n*-6 LCPUFA content was higher in the placebo group than in the supplemented groups (data not shown) 24.8 ± 0.23 % and 22.65 ± 0.31% respectively (*p *< 0.0001).

### Alteration in leukocyte levels

**Figure 2:**
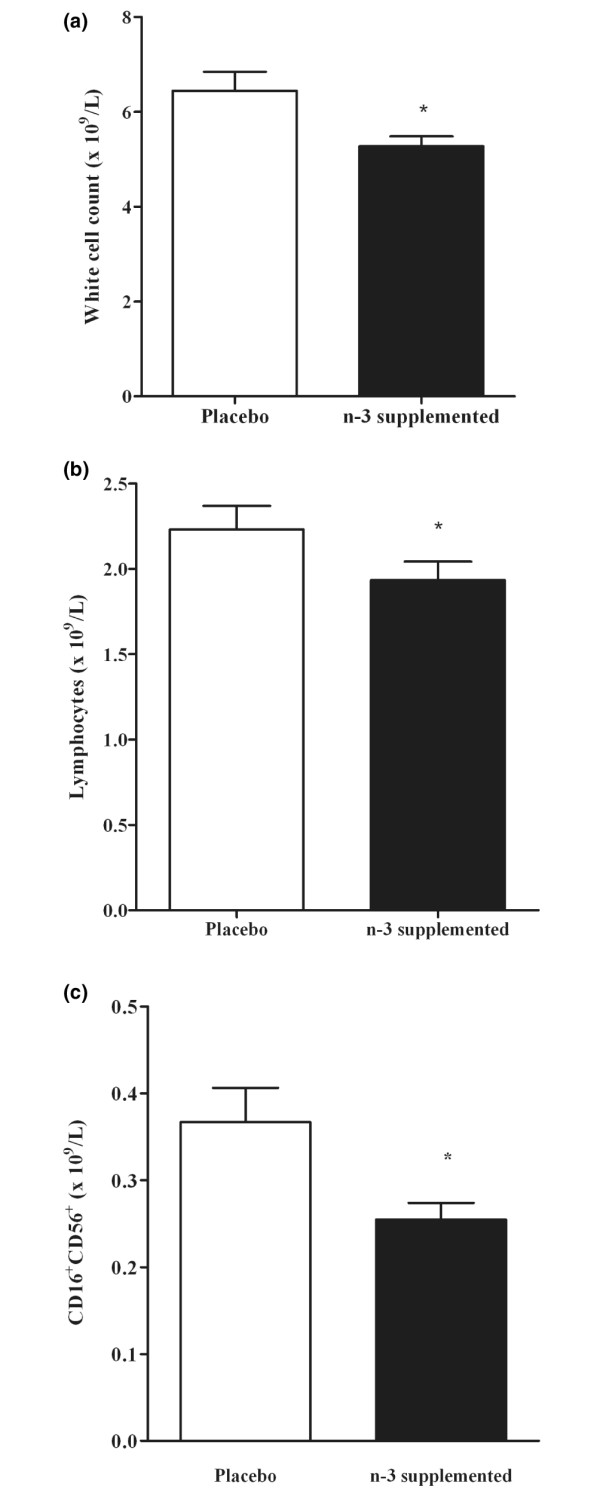
Effect of *n*-3 LCPUFA supplementation on leukocyte numbers. Number of **(a) **total white cells, **(b) **lymphocytes and **(c) **NK cells (CD16^+^CD56^+^) for the placebo and *n*-3 supplemented groups. Blood was taken after 6 months and white-cell enumeration carried out as outlined in Materials and methods. Data are expressed as mean ± SEM. *p < 0.05, significance of difference between placebo and supplemented (Student *t*-test). *n *= 19 for both placebo and supplemented groups.

Total leukocyte levels were significantly lower in *n*-3-supplemented subjects than in controls (Figure 2a). This difference was reflected in total lymphocytes (Figure 2b). Linear regression for lymphocyte numbers versus percentage of plasma membrane fatty acid showed a significant negative correlation with EPA levels (Table 1). The correlation with DHA and total *n*-3 LCPUFA, though not significant, showed the same trend of decreasing lymphocyte numbers with higher membrane *n*-3 LCPUFA content.

**Table 1:** Regression analysis of membrane *n*-3 LCPUFA content versus lymphocyte subpopulation^a ^numbers and cytokine production

	EPA		DHA		*n*-3 LCPUFA^c^	
	
	*r*^b^	*p *value^b^	*r*	*p *value	*r*	*p *value
CD3^+^	-0.32	0.07	-0.06	0.74	-0.20	0.27
CD4^+^	-0.34	0.06	-0.04	0.84	-0.19	0.28
CD8^+^	-0.17	0.36	-0.07	0.70	-0.12	0.50
CD4^+^:CD8^+^	0.14	0.45	0.01	0.94	0.06	0.73
CD16^+^CD56^+^	**-0.39^d^**	**0.025**	**-0.37**	**0.033**	**-0.38**	**0.029**
CD19^+^	-0.13	0.47	-0.11	0.56	-0.12	0.50
IFNγ	0.17	0.43	0.13	0.56	0.16	0.50
IL-2	0.23	0.30	0.23	0.30	0.26	0.24
LT	**0.43**	**0.046**	0.18	0.44	0.27	0.22
Lymphocytes	**-0.45**	**0.009**	-0.21	0.25	-0.28	0.11
Neutrophils	-0.24	0.18	-0.31	0.065	-0.31	0.078
Monocytes	0.06	0.80	0.41	0.11	0.28	0.27
Total leukocytes	-0.33	0.06	-0.32	0.07	-0.34	0.06

^a^CD3^+^, all circulating T cells; CD4^+^, all T helper cells; CD8^+^, all cytotoxic T cells; CD19^+^, all B lymphocytes; CD16^+^CD56^+^, all NK cells; IFNγ, interferon-gamma; IL-2, interleukin 2; LT, lymphotoxin. ^b^The correlation coefficient *r *value; the *p *value shows the significance (*p *< 0.05) (ANOVA) of the obtained *r *value. ^c^*n*-3 LCPUFA refers to the sum of docosapentaenoic acid (DPA), eicosapentaenoic acid (EPA) and docosahexaenoic acid (DHA). ^d^Boldfaced text shows results with *r *value of *p *< 0.05.

**Figure 3:**
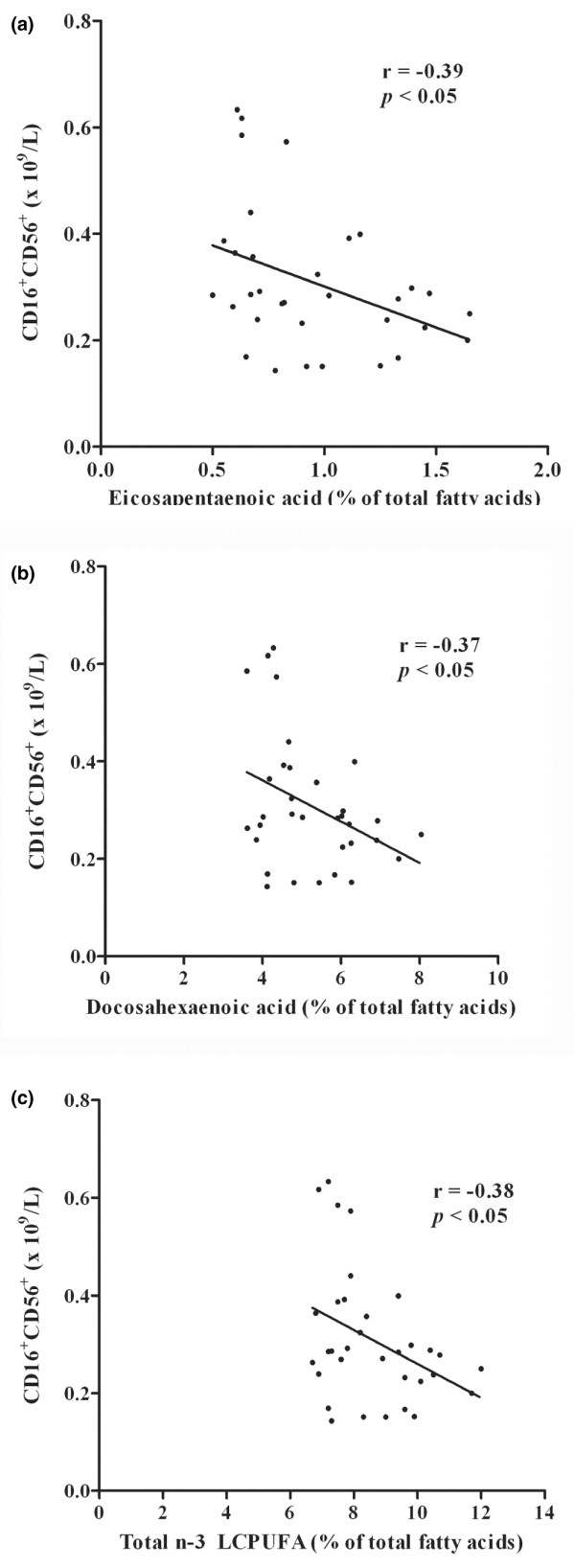
Relationship between NK-cell numbers and erythrocyte membrane lipids. **(a) **Eicosapentaenoic acid (EPA), **(b) **docosahexaenoic acid (DHA), and **(c) **total *n*-3 LCPUFA (EPA + DPA + DHA) after 6 months of supplementation; *n *= 33. CD16^+^CD56^+^, NK cells.

Comparing the data between the *n*-3 LCPUFA supplemented and the placebo group with respect to levels of other leukocyte subpopulations showed that there was no significant difference in CD3^+^, CD4^+^, CD8^+^, and CD19^+ ^cells (Table 2). Similarly there was no significant negative correlation between the amount of membrane *n*-3 LCPUFA, EPA and DHA in erythrocytes and the levels of these leukocyte subpopulations (Table 1). However, the number of NK cells was significantly lower with *n*-3 LCPUFA supplementation (Figure 2c). Regression analysis showed a significant negative correlation between the number of CD16^+^/CD56^+ ^cells and amounts of EPA, DHA and total *n*-3 LCPUFA (Figure 3). The data show that with higher *n*-3 PUFA content there are fewer NK cells. In comparison, when NK cells were correlated with the total amount of *n*-6 PUFA, which decreased in the supplemented group compared with the placebo, as demonstrated above, there was a positive correlation (*r *= 0.34; *p *< 0.05), higher amounts of total *n*-6 PUFA were associated with higher NK-cell levels (data not shown).

**Table 2:** Effect of 6 months of supplementation with *n*-3 enriched foods on absolute numbers of other leukocyte subpopulations

Group^a^	CD3	CD4	CD8	CD19	Neutrophils	Monocytes
Placebo	1.64 ± 0.12^b^	1.05 ± 0.09	0.59 ± 0.07	0.21 ± 0.02	3.51 ± 0.19	0.25 ± 0.03
*n*-3 supplemented	1.47 ± 0.09	0.95 ± 0.07	0.53 ± 0.04	0.18 ± 0.02	3.04 ± 0.13	0.22 ± 0.02

^a^CD3, all circulating T-cells; CD4, all T helper cells; CD8, all cytotoxic T cells; CD19, all B lymphocytes. ^b^Data are presented as cells/l (× 10^9^) mean ± SEM (*n *= 16 to 17).

### Lymphocyte function

**Figure 4:**
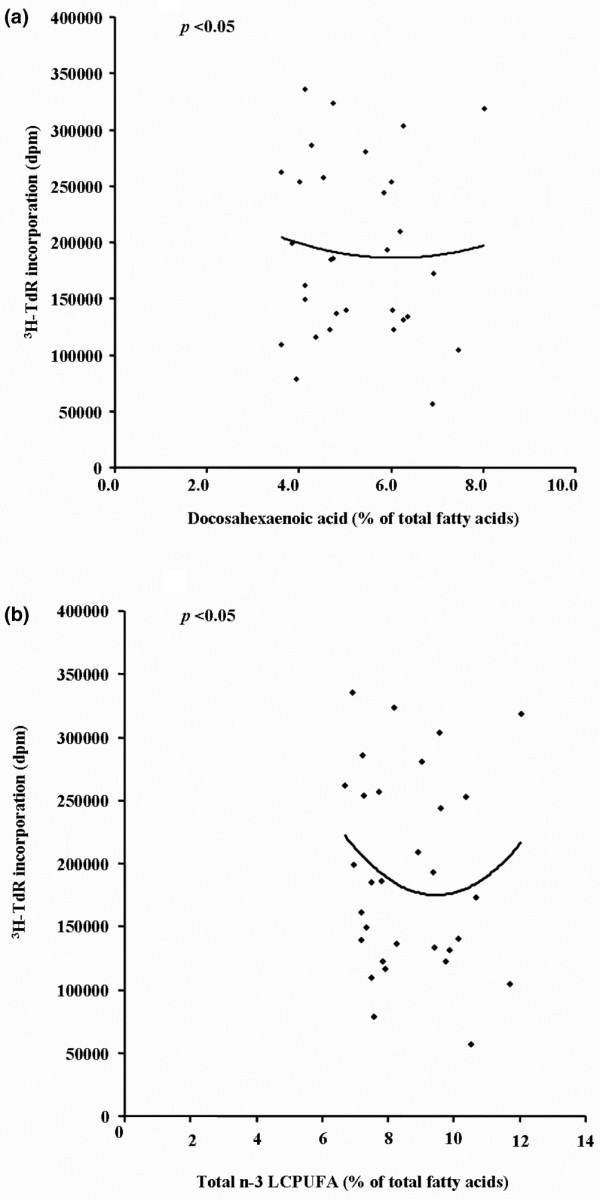
Relationship between PHA-induced proliferation in peripheral blood mononuclear cells and the *n*-3 LCPUFA content of erythrocyte membranes. Proliferation was measured by incorporation of [^3^H]thymidine (^3^H-TdR). There was a significant correlation between [^3^H]thymidine incoporation and **(a) **DHA and **(b) **total *n*-3 LCPUFA (EPA + DPA + DHA) following a curve (*p *< 0.05; *n *= 31).

There was no difference in the PHA-induced lymphocyte proliferation in MNL from the *n*-3 LCPUFA-supplemented and placebo groups (Table 3). However, when relating the proliferation response to the levels of *n*-3 LCPUFA in the plasma membrane lipids, we demonstrated that the relationship follows a curve (U-shaped) rather than a line (Figure 4). Thus with higher membrane *n*-3 LCPUFA levels, PHA-induced proliferation was initially lower but with further increases in *n*-3 LCPUFA content proliferation increased.

**Table 3:** Effect of 6 months of supplementation with *n*-3 enriched foods on PHA-induced cytokine production and proliferation in peripheral blood mononuclear leukocytes

Group	IL-2^a^	IFNγ^a^	PHA proliferation^b^
Placebo	1.83 ± 0.61	39.77 ± 12.53	193800 ± 20840
*n*-3 supplemented	4.01 ± 1.73	57.24 ± 11.62	191100 ± 19520

IL-2, interleukin 2; IFNγ, interferon-gamma; PHA, phytohaemagglutinin. ^a^Cytokine data are presented as ng/ml, mean ± SEM (*n *= 9 to 13). ^b^Proliferation data expressed in disintegration per minute (dpm).

**Figure 5:**
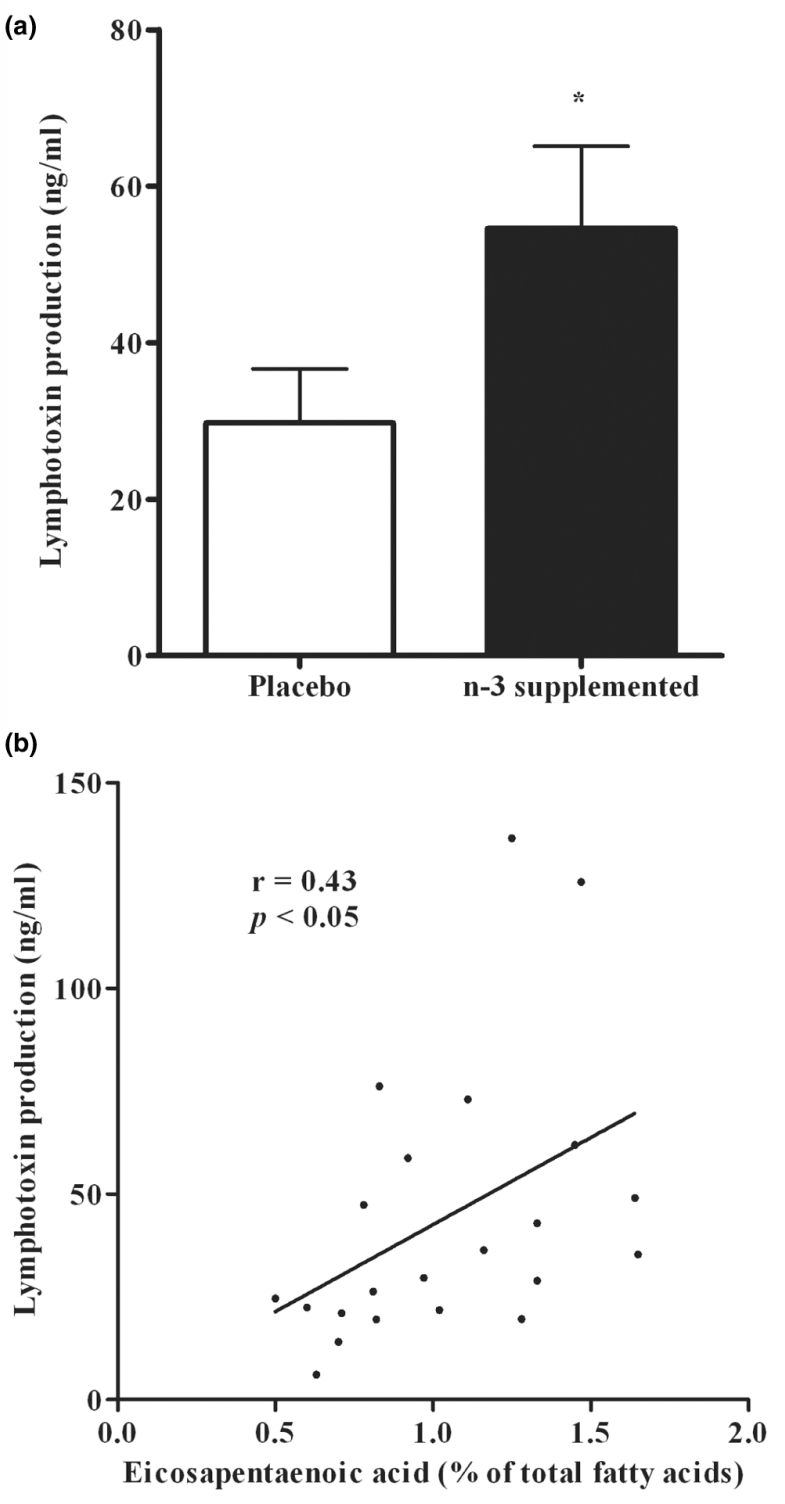
Effect of *n*-3 LCPUFA supplementation on the production of lymphotoxin (LT). **(a) **Peripheral blood mononuclear leukocytes were incubated with PHA and the production of LT was determined after 72 h incubation. *p < 0.05, significance of difference between placebo and supplemented (Student *t*-test). Data are expressed as mean ± SEM; *n *= 9 for the placebo group and *n *= 13 for the supplemented group. **(b) **Relationship between LT production and erythrocyte membrane eicosapentaenoic acid (EPA) after 6 months supplementation.

The production of IFNγ and IL-2 by MNL stimulated by PHA was not significantly different between the *n*-3 LCPUFA supplemented and placebo groups (Table 3). Furthermore, there was no correlation between the membrane *n*-3 LCPUFA and ability to produce cytokine (Table 1). In contrast, LT production by PHA-stimulated MNL was much greater in the *n*-3-supplemented group compared with placebo (Figure 5) and showed a significant and positive correlation with EPA erythrocyte content (Table 1, Figure 5).

### Neutrophil functions

**Figure 6:**
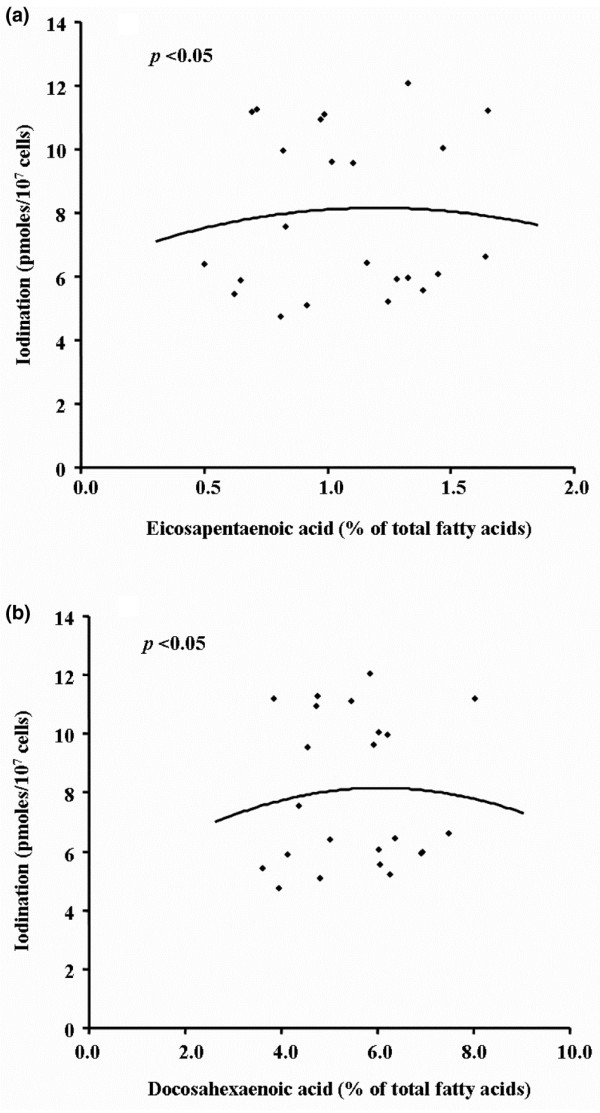
Relationship between neutrophil quantitative iodination reaction and the *n*-3 LCPUFA content of erythrocyte membranes. There was a significant correlation between the iodination activity and **(a) **EPA and **(b) **DHA following a curve (*p *< 0.05; *n *= 23).

When comparing the neutrophil functional responses between *n*-3 LCPUFA-supplemented subjects and placebo, we found no significant differences in chemotaxis, chemokinesis, adherence and bactericidal activity (Table 4), and no significant differences between the *n*-3 LCPUFA levels in the erythrocyte membrane and all these functions, apart from the neutrophil iodination reaction (Table 5). The iodination response showed a 'bell-shaped' relationship when analyzed against the EPA and DHA content of erythrocyte membranes (Figure 6). This shows that there is greater iodination reaction with higher *n*-3 LCPUFA content. However, with further increases in *n*-3 LCPUFA levels, the neutrophil iodination activity was lower.

**Table 4:** Effect of 6 months of supplementation with *n*-3 enriched foods on neutrophil functions

Group	Adherence^a^	Bactericidal^b^	Chemokinesis^c^	Chemotaxis^c^	Iodination^d^
Placebo	0.36 ± 0.10	95.4± 0.52	0.76 ± 0.09	1.94 ± 0.092	7.93 ± 0.98
*n*-3 supplemented	0.29 ± 0.058	97.1 ± 0.63	0.76 ± 0.0356	1.91 ± 0.07	8.03 ± 0.65

Data are presented as mean ± SEM (*n *= 16 to 17). ^a^Adherence expressed as relative absorbance at 570 nm.

^b^Bactericidal activity expressed as % killing. ^c^Chemokinesis and chemotaxis expressed in mm/90 min. ^d^Iodination expressed in pmoles iodine/10^7 ^cells.

**Table 5:** Regression analysis of membrane *n*-3 LCPUFA content versus neutrophil function

Function	EPA		DHA		*n*-3 LCPUFA^a^	
	
	*r*^b^	*p *value^b^	*r*	*p *value	*r*	*p *value
Adherence	-0.09	0.73	-0.08	0.77	-0.072	0.79
Chemotaxis	-0.11	0.62	-0.12	0.57	-0.12	0.58
Chemokinesis	0.20	0.38	0.13	0.55	0.23	0.30
Bactericidal	0.30	0.15	0.21	0.33	0.25	0.24
Iodination	0.04	0.86	0.04	0.86	0.01	0.95

^a^*n*-3 LCPUFA refers to the sum of docosapentaenoic acid (DPA), eicosapentaenoic acid (EPA) and docosahexaenoic acid (DHA). ^b^The correlation coefficient *r *value; the *p *value shows the significance (*p *< 0.05) (ANOVA) of the obtained *r *value.

## Discussion

The data showed that in association with an increase in consumption of *n*-3 LCPUFA there was a significant reduction in levels of circulating NK cells (CD16^+ ^CD56^+^). Comparisons between the supplemented and placebo group indicate that *n*-3 LCPUFA reduces NK-cell numbers in the circulation. Analysis of the NK-cell levels against the amount of *n*-3 LCPUFA in the erythrocyte membranes established a negative correlation with the level of EPA, DHA and total *n*-3 LCPUFA. A linear correlation was seen over the *n*-3 LCPUFA range of 6.68 to 12.05%. A similar correlation was seen over the EPA range of 0.48 to 1.65% and DHA of 3.61 to 8.04%. The reduced NK-cell numbers can only be contributing in a very small way to the reduced levels of total leukocytes and lymphocytes seen over this range of *n*-3 LCPUFA, EPA and DHA levels. While we also observed lower levels of neutrophils, T cells, B cells and T-cell subpopulations, this was not significant. This suggests that NK cells are most sensitive to increasing amounts of *n*-3 LCPUFA. Previously, others have reported that *n*-3 PUFA supplementation leads to reduced NK activity. Thies *et al*. [19] reported that only a moderate intake of EPA + DHA (720 mg + 280 mg)/day (but not DHA alone), α-linolenic (18:3n-3) or γ-linolenic acid (18:3n-6) over a 12-week period caused a reduction in NK-cell activity. No corresponding decrease in numbers or proportions of NK cells was observed. As our studies demonstrated that reduced levels are associated with greater amounts of *n*-3 LCPUFA in cell membranes, it is likely that the difference is related to the duration of the supplementation period. In fact our observations at 12 weeks of supplementation revealed that NK-cell numbers were not significantly correlated with *n*-3 LCPUFA levels in erythrocyte membranes (data not shown). Kelley *et al*. [20] reported a reduction in NK-cell activity using 6 g DHA/day over 12 weeks and this was also associated with an increase in DHA (2.3 to 7.4% of total fatty acids) in MNL. However, the NK-cell numbers or proportions were not measured. More recently, Miles *et al*. [21] reported that supplementation with 4 g/day of EPA had little effect on the magnitude of NK-cell activity. We now report that a major consequence of increasing cell membrane levels of *n*-3 LCPUFA is a correlated decrease in NK-cell numbers. Collectively, the above studies suggest that under appropriate *n*-3 PUFA enrichment, both NK-cell numbers and NK-cell activity are reduced.

While NK cells are important in immune surveillance, particularly against viral infections and cancer [22-24], the cells are also likely to play a role in the pathogenesis of inflammatory diseases as they have the ability to produce high levels of the pro-inflammatory cytokines IFN-γ, IL-1 and TNF, and are cytotoxic for tissues. For example, decreased NK-cell activity has been implicated as a protective mechanism of *n*-3 LCPUFA (EPA and DHA) in patients with ulcerative colitis [25]. Aaskov *et al*. [26] reported that NK cells from patients with epidemic polyarthritis could lyse autologous synovial cells and hence contribute to the arthritis. The ability of *n*-3 LCPUFA to reduce circulating NK-cell numbers could be another way in which *n*-3 LCPUFA protect against these inflammatory disorders, acting in concert with the established dogma that *n*-3 LCPUFA reduce the levels of potent pro-inflammatory eicosanoids [9]. Our findings also raise the possibility that *n*-3 LCPUFA may protect during infection-induced exacerbation of tissue damage in RA patients through an effect on NK-cell levels. Whether or not the NK cells in our studies were also less cytotoxic remains to be established, but on the basis of previous reports this is likely and would add to the anti-inflammatory effects, which our findings support. The basis for the reduced NK-cell numbers will need to be further studied. However, it is likely to be related to an effect on hematopoiesis, perhaps selectively affecting NK-cell development.

Earlier studies, which examined the effects of dietary supplementation with *n*-3 LCPUFA on immune function, in most cases used daily doses as high as 5.4 g and, not surprisingly, demonstrated a marked suppression of immunological responses such as neutrophil chemotaxis, MNL cytokine production and lymphocyte proliferation [27-29]. Another study, which used high doses of *n*-3 LCPUFA, (14.4 g per day over 10 weeks) reported a large reduction in neutrophil chemotaxis (93%) [27]. It is usually accepted that depressed neutrophil functions are not seen at lower doses such as those used in our study.

At these lower doses we found very little effect on lymphocyte function. There was no difference in lymphocyte proliferation to PHA between the *n*-3 LCPUFA-supplemented and placebo groups. This contrasted with the observations of the production of cytokines by T cells. Whereas there was no significant difference between the two groups in the production of IL-2 and IFNγ, LT production was increased in the *n*-3 LCPUFA-supplemented group compared with placebo at 6 months. However, the PHA-induced proliferation exhibited a U-shaped relationship with the increases in *n*-3 LCPUFA content. This effect of PHA-induced proliferation was somewhat mirrored in the production of PHA-induced LT. Regression analysis showed a positive correlation between the LCPUFA levels in erythrocyte membranes and LT production by lymphocytes. The discrepancy in the pattern of regression – that is, linear in the case of LT or U-shaped in that of PHA-induced proliferation – suggests that the proliferation response is more complex in terms of sensitivity to the membrane content of fatty acids compared to the production of LT. It remains to be established why low-dose *n*-3 LCPUFA supplementation promotes production of LT but not the other T-cell cytokines, IFNγ and IL-2. Nevertheless, such increases in LT production can be protective against tissue damage in RA. It has been demonstrated that, unlike TNF, IFNγ and IL-2, which augment neutrophil-mediated inhibition of cartilage proteoglycan synthesis, LT markedly depressed this activity [30]. Others have shown that with increases in dose of EPA there is greater production of the anti-inflammatory cytokine IL-4 [21].

Neutrophil functions, chemotaxis, chemokinesis, adhesion and bactericidal activity were unaffected by *n*-3 LCPUFA supplementation. Our finding of bell-shaped relationships for the iodination activity may also be of importance in protection against tissue damage in RA. The iodination reaction is a measure of the production of hypochlorous acid by neutrophils through the generation of H_2_O_2 _and release of myeloperoxidase [31]. Interestingly this system was found to be important for articular cartilage damage by neutrophils [30].

Analyses of the relationship between membrane LCPUFA profiles and the content and immune function of peripheral blood leukocytes has been previous examined in non-supplemented subjects [32], who would be expected to have lower *n*-3 LCPUFA levels than our study subjects who received *n*-3 LCPUFA supplementation. Kew *et al*. [32] reported that DHA was positively correlated with neutrophil, monocyte and lymphocyte responsiveness. This relationship was maintained even with AA, although there was a negative correlation with the *n*-6:*n*-3 ratio. This may be different when the higher levels attained with *n*-3 supplementation are taken into consideration. As Kew *et al*. [32] indicated in their studies, there might be a bell-shaped relationship between functional activity of leukocytes and LCPUFA concentration. Indeed, this was evident in the current study where *n*-3 supplementation was used. Others have reported a U-shaped correlation between doses of fish oil supplementation (0.3, 1.0 and 2.0 g/day) and leukocyte function [33].

Our approach was to analyze the immune parameters against the corresponding LCPUFA level in erythrocyte membrane lipids of the individual subject. These levels can be related to supplementation doses and also serve to overcome some limitations associated with compliance and various confounding factors. It is also apparent that *n*-3 LCPUFA levels in plasma, erythrocytes and leukocytes can be correlated. Thus changes in levels of EPA and DHA in erythrocytes and leukocytes appear to correlate during *n*-3 LCPUFA supplementation [34,35], although the rate of incorporation between these cell types is different in the first 6 to 12 weeks of supplementation. Leukocytes, especially neutrophils, are likely to undergo some activation or stimulation during purification and thus the fatty-acid content may not be a reflection of the amount in the unactivated state, possibly explaining some of the controversies in findings by different laboratories. Thus the measurements in erythrocytes may be a more reliable marker for routine testing than those in leukocytes, although inevitably the latter are required to delineate the mechanisms involved. While future studies should consider the relationship between the cell type being examined and levels within that cell, this then becomes a complex question, as leukocytes consist of subpopulations of different cell types.

While the lower number of NK cells associated with low-dose long-term *n*-3LCPUFA supplementation is likely to be beneficial to patients with inflammatory disorders, it is also evident that individuals consuming these fatty acids may have greater susceptibility to viral infections. NK cells provide a first-line defense against these pathogens [36]. It is, however, reassuring that at these lower doses of supplementation, most components of the innate and adaptive immune response were not affected over the long term.

## Conclusion

The approach reported here illustrates the importance of relating immune parameters to the levels of erythrocyte membrane PUFA composition to establish a biochemical basis for endeavors to supplement people with *n*-3 LCPUFA using functional foods. It is thus tempting to suggest that such measurements could be used as indices for predictions of protection against inflammation, and as a way of controlling the level of *n*-3 LCPUFA supplementation. Furthermore, of all the immune parameters measured, NK cells were the most sensitive to the effects of increasing amounts of *n*-3 LCPUFA in the diet. This is conducive to *n*-3 LCPUFA supplementation reducing cellular inflammation and tissue damage in diseases such as RA. Future studies designed to elucidate the basis for the reduced numbers of NK cells could give us new directions for the use of *n*-3 LCPUFA supplementation.

## Abbreviations

*n*-3 LCPUFA = omega-3 long-chain polyunsaturated fatty acids; AA = arachidonic acid; EPA = eicosapentaenoic acid; DHA = docosahexaenoic acid; MNL = mononuclear cells; NK cells = natural killer cells.

## Competing interests

The authors declare that they have no competing interests.

## Authors' contributions

VRM was responsible for conducting the immunology experiments; KJM was responsible for conducting the fatty-acid analyses. PRH and KJM were responsible for the organization of the subjects and diet supplements. All authors participated in study design, writing the manuscript and data analyses/interpretation.
